# *Helicobacter pylori* Vacuolating Toxin and Gastric Cancer

**DOI:** 10.3390/toxins9100316

**Published:** 2017-10-12

**Authors:** Mark S. McClain, Amber C. Beckett, Timothy L. Cover

**Affiliations:** 1 Department of Medicine, Vanderbilt University School of Medicine, Nashville, TN 37232, USA; mark.s.mcclain@vanderbilt.edu; 2 Department of Pathology, Microbiology and Immunology, Vanderbilt University Medical Center, Nashville, TN 37232, USA; amber.beckett@vanderbilt.edu; 3 Veterans Affairs Tennessee Valley Healthcare System, Nashville, TN 37212, USA

**Keywords:** *Helicobacter pylori*, gastric cancer, pore-forming toxins

## Abstract

*Helicobacter pylori* VacA is a channel-forming toxin unrelated to other known bacterial toxins. Most *H. pylori* strains contain a *vacA* gene, but there is marked variation among strains in VacA toxin activity. This variation is attributable to strain-specific variations in VacA amino acid sequences, as well as variations in the levels of VacA transcription and secretion. In this review, we discuss epidemiologic studies showing an association between specific *vacA* allelic types and gastric cancer, as well as studies that have used animal models to investigate VacA activities relevant to gastric cancer. We also discuss the mechanisms by which VacA-induced cellular alterations may contribute to the pathogenesis of gastric cancer.

## 1. Description of VacA

*H. pylori* VacA derives its name from the protein’s ability to induce vacuolation in intoxicated cells. Vacuolation of epithelial cells was the first reported effect of VacA [1,2], but many other cellular effects have been reported subsequently, and many cell types are now known to be susceptible to the toxin [3,4,5,6]. The effects of VacA on gastric epithelial cells include cytoplasmic vacuolation [7,8], disrupted endocytic trafficking, mitochondrial perturbations, depolarization of the plasma membrane potential, efflux of various ions (including chloride, bicarbonate, and urea), activation of MAP kinases, modulation of autophagy, and potentially cell death [3,4,5,6,9]. VacA can inhibit the function and proliferation of a variety of immune cells, including T cells, B cells, eosinophils, macrophages, dendritic cells, and neutrophils [3,4,5,6,10,11].

The amino acid sequence and structure of VacA are unrelated to the sequences or structures of other known bacterial toxins [12,13,14,15]. VacA is produced as a 140 kDa precursor, which undergoes proteolytic processing to yield an 88 kDa toxin [2,16,17,18,19]. An amino-terminal signal peptide and a carboxy-terminal domain are required for export of the toxin into the extracellular space through a type V (autotransporter) secretion pathway [16,17,20,21,22]. The 88 kDa VacA toxin can undergo further proteolytic cleavage, resulting in amino-terminal 33 kDa (p33) and carboxy-terminal 55 kDa (p55) fragments [18,23,24,25], but there is no evidence that this cleavage is required for the toxin’s activities [26]. Both the p33 and p55 domains are important for toxin binding to cells and internalization of the toxin into mammalian cells [27,28]. Experiments analyzing VacA fragments expressed in transfected mammalian cells revealed that the minimum-length fragment required to induce vacuolation includes the entire p33 domain plus the amino-terminal ~110 amino acids of the p55 domain [29,30,31].

VacA binds to the surface of cells within lipid rafts, corresponding to detergent-resistant membrane fractions [32,33,34]. Multiple VacA receptors have been reported, including sphingomyelin, receptor-like protein tyrosine phosphatase alpha (RPTP-α), RPTP-β, and low density lipoprotein receptor-related protein-1 (LRP-1) on epithelial cells [35,36,37], and β2 integrin (CD18) on T cells [38]. After binding to the cell surface, VacA is subsequently internalized into endosomal compartments [39,40,41,42,43,44]. Internalized VacA associates not only with endosomal compartments, but has also been reported to associate with mitochondria [45,46,47,48], the Golgi apparatus, and endoplasmic reticulum [49]. VacA is not known to possess an enzymatic activity, but it can undergo insertion into membranes to form anion-selective channels [50,51,52,53,54,55,56,57,58]. VacA forms channels in the plasma membrane [53,55], and channels are also presumed to form within endosomal membranes of mammalian cells.

The membranes of VacA-induced vacuoles contain markers of late endosomes and lysosomes [44,49,59,60], suggesting that VacA-induced vacuoles are derived from the endosome-lysosome pathway. It has been proposed that the formation of VacA anion channels in endosomal membranes, coupled with vacuolar ATPase activity, leads to the osmotic swelling of endosomal compartments and the formation of vacuoles visible by light microscopy [40,61,62]. VacA-induced alterations in endocytic processes or intracellular trafficking result in inhibited intracellular degradation of epidermal growth factor (EGF), inhibited maturation of procathepsin D, perturbation of transferrin receptor localization, and inhibition of antigen presentation [63,64,65]. VacA’s association with mitochondria can lead to decreased mitochondrial membrane potential, the activation of BAX and BAK, cytochrome c release, and mitochondrial fragmentation [45,46,47,48,66,67,68]. Mitochondrial perturbation by VacA is dependent on VacA channel activity [46,47] and contributes to cell death through apoptosis or necrosis [48,69,70,71,72]. VacA-induced cell death may also be a consequence of the reduced expression of pro-survival factors [73].

## 2. Heterogeneity among *vacA* Alleles

**Figure 1:**
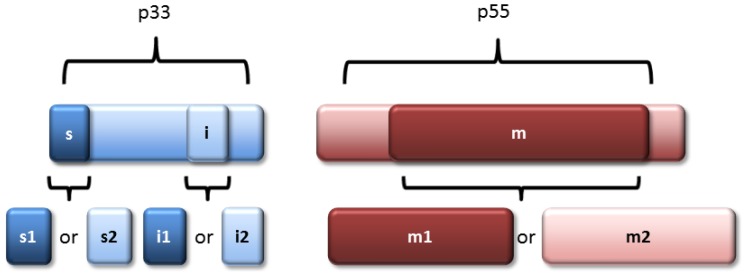
*vacA* allelic diversity. Three main regions of *vacA* heterogeneity are recognized, designated as the signal or “s” region, the intermediate or “i” region, and the middle or “m” region. The sequences in each of these regions can be classified into two main families (s1 and s2; i1 and i2; m1 and m2). The figure illustrates the relationship of these regions to VacA p33 and p55 domains.

All *H. pylori* strains contain a *vacA* gene, but there is substantial variation among strains in VacA toxin activity. A lack of vacuolating toxin activity occasionally results from nonsense mutations or frameshift mutations in *vacA* [74], but this is a relatively uncommon phenomenon; most strains contain intact *vacA* ORFs. Among strains containing an intact *vacA* ORF, differences in VacA toxin activity are attributable to variations in VacA amino acid sequences [75,76,77,78,79], as well as differences among strains in the levels of VacA transcription or secretion [80]. The *vacA* alleles in different *H. pylori* strains have been categorized into several families, based on sequence heterogeneity in specific regions. The three most extensively studied regions of heterogeneity correspond to the signal or “s” region, the intermediate or “i” region, and the middle or “m” region [75,81]. The sequences in each of these regions can be classified into two main families (e.g., s1 and s2; i1 and i2; m1 and m2) (Figure 1). *vacA* alleles have also been classified into two families (d1 and d2) based on the presence or absence of a segment ranging from about 60 to 100 nucleotides in length, designated the d-region [82], which encodes a region of VacA located at the junction of the p33 and p55 domains.

The “s” region of diversity corresponds to sequence differences within the amino-terminal signal peptide and the amino–terminal end of the secreted toxin. Compared with s1 VacA toxins, s2 forms of VacA contain a 12-amino-acid amino-terminal extension that alters the hydrophobicity of the amino-terminal end of the secreted protein [75,76,77,78]. In comparison to s1 VacA toxins, s2 VacA toxins are impaired in terms of their ability to form anion channels in planar-lipid bilayers and do not cause vacuolation of mammalian cells [75,76,77,78]. Type s2 forms of *vacA* are also transcribed at lower levels than type s1 forms, resulting in reduced levels of type s2 VacA protein production and secretion [80].

The “i” region of diversity is located within the p33 domain of VacA [81]. One study reported that the i-region is a determinant of vacuolating toxin activity in strains that produce type s1-m2 forms of VacA [81]. Type i1 VacA toxins are also more active than i2 VacA toxins in assays monitoring the inhibition of NFAT activation and IL-2 production by Jurkat T cells [83].

Finally, the “m” region of diversity is located within the p55 domain of VacA [75]. In comparison to type m2 VacA proteins, type m1 VacA proteins have greater vacuolating activity on HeLa cells, but m1 and m2 VacA proteins have similar vacuolating activity on RK13 cells [84,85,86,87]. A region responsible for cell type specificity is localized to a 148 amino-acid segment of the m region [85,86]. The difference in HeLa cell vacuolating activity when comparing m1 and m2 VacA proteins has been attributed to differences in channel-forming properties [88], as well as differences in cell-binding properties [84,86]. Type m1 VacA, but not m2 VacA, binds to the LRP1 receptor on host cells, resulting in decreased levels of intracellular glutathione, an accumulation of reactive oxygen species, autophagy, and apoptosis [89,90].

*H. pylori* is naturally competent for the uptake of DNA and intraspecies recombination commonly occurs. Therefore, *vacA* alleles with nearly all combinations of s-, i-, and m-regions (s1-i1-m1, s1-i1-m2, s1-i2-m2, s2-i2-m2, etc.) have been detected, as well as chimeric i-regions (e.g., i1-i2) and chimeric m-regions (e.g., m1-m2) [75,79,91,92,93]. Notably, *vacA* alleles with an s2-i1-m1 organization are uncommon [75,94], which suggests that the activity of such proteins is either detrimental or confers less benefit to the bacteria than other types of VacA proteins.

## 3. *vacA* Allelic Types and Gastric Cancer Risk

There has been considerable interest in the possibility that the VacA toxin activity of strains might be a determinant of gastric cancer risk [95,96,97]. To test this hypothesis, *H. pylori* strains cultured from individuals with gastric cancer or premalignant gastric pathology (such as atrophic gastritis, intestinal metaplasia, or dysplasia) have been compared to strains cultured from individuals with non-malignant gastric histology. Collectively, these studies have shown that strains containing type s1, i1, and m1 *vacA* alleles are associated with a higher risk of gastric cancer or premalignant conditions, compared to strains containing type s2, i2, or m2 *vacA* alleles, respectively [81,98,99,100,101,102,103,104,105,106,107]. Strains containing type s1 and m1 *vacA* alleles have also been associated with an increased severity of gastric inflammation, epithelial damage, or ulceration, compared to strains containing type s2 or m2 *vacA* alleles (Table 1) [75,108,109,110]. Thus, strains encoding forms of VacA with greater activity in cell culture models are associated with an increased risk of gastric cancer and premalignant histologic changes, as well as an increased risk of peptic ulceration, compared to strains encoding forms of VacA that lack activity or have relatively low levels of activity in cell culture models.

## 4. Association between *vacA* Allelic Types and Other Strain-Specific Virulence Determinants of Virulence

In addition to allelic variation in *vacA, H. pylori* strains exhibit diversity in other genetic elements that are relevant for gastric cancer pathogenesis. One of the most prominent genetic variations among *H. pylori* strains is the presence or absence of a ~40 kb chromosomal region known as the *cag* pathogenicity island (PAI). The *cag* PAI encodes an effector protein (CagA), as well as components of a type IV secretion system that delivers CagA into host cells [111,112,113]. Upon entry into epithelial cells, CagA interacts with multiple host cell proteins and causes alterations in cell signaling [114,115]. *H. pylori* strains also differ in the production of outer membrane proteins (OMPs), including adhesins that mediate adhesion to gastric epithelial cells. Examples of adhesins that are produced by some *H. pylori* strains but not others include BabA, SabA, and HopQ [116,117].

*H. pylori cagA*-positive strains (corresponding to strains that contain the *cag* PAI) are associated with a higher risk of gastric cancer or premalignant lesions than *cagA*-negative strains [98,118,119]. Similarly, *H. pylori* strains containing specific OMP-encoding genes (*babA, homB,* type I *hopQ,* in-frame *hopH/oipA*, or in-frame *sabA* alleles) are associated with an increased risk of gastric cancer or premalignant changes compared to strains that lack these genes or that harbor out-of-frame genes [120,121,122,123,124,125,126].

*vacA* alleles, the *cag* PAI, and several genes encoding strain-specific OMPs are not distributed randomly among *H. pylori* strains [117]. For example, strains containing type s1 *vacA* harbor the *cag* PAI more commonly than strains containing s2 *vacA* alleles [75,109]. Strains containing type s1 *vacA* also contain the OMP-encoding genes *babA, homB* type I *hopQ*, and in-frame *hopH/oipA* more commonly than strains that contain type s2 *vacA* [75,110,116,120,127,128,129,130,131]. Several studies have reported that VacA and CagA have reciprocal antagonistic effects [71,132,133,134,135,136]. Thus, certain combinations of *vacA* and *cagA* alleles may confer a selective advantage to strains by offering an optimal balance of VacA and CagA activities.

Determining the specific contribution of VacA to gastric cancer risk is challenging, since the strains associated with gastric cancer potentially contain multiple strain-specific features relevant for gastric cancer pathogenesis. Collectively, the epidemiologic studies suggest that the risk of gastric cancer is highest in persons infected with strains producing multiple host-interactive components (type s1-i1-m1 VacA, CagA, the *cag* T4SS, and certain strain-specific OMPs) [98,117,120]. Strains that do not produce these components are associated with a lower level of gastric cancer risk.

Multiple *vacA* allelic types (s1 or s2, i1 or i2, m1 and m2) are present in *H. pylori* isolates in Western countries [75,81], and both *cag* PAI-positive strains and *cag* PAI-negative strains are common in Western countries [110]. In contrast, nearly all *H. pylori* strains cultured in several regions of East Asia, including Japan and Korea, contain s1 *vacA* alleles [137,138], and nearly all *H. pylori* strains in Japan and Korea contain the *cag* PAI [110,137]. Strains containing type s2 *vacA* alleles and lacking the *cag* PAI are relatively uncommon in East Asia [110,137,138]. These characteristics of East Asian strains may be an important factor contributing to the high rate of gastric cancer in East Asia compared to many other parts of the world [139].

## 5. Impact of VacA on *H. pylori* Gastric Colonization of Animal Models

Nearly all *H. pylori* strains contain an intact *vacA* ORF, which suggests that VacA has an important role in *H. pylori* colonization of the stomach, persistence, or transmission to new hosts. Several studies have evaluated the role of VacA in *H. pylori* colonization of animal models by testing *vacA* null mutant strains. Such mutant strains are capable of colonizing the stomach in gnotobiotic piglet, mouse, and gerbil models [107,140,141,142,143,144]. Moreover, several closely related *H. pylori* strains (strains B128, B8 and 7.13) capable of colonizing the Mongolian gerbil do not produce a detectable VacA protein due to the presence of a naturally occurring mutation in *vacA* [145,146,147]. Although VacA is not essential for *H. pylori* colonization of the stomach in animal models, *vacA* mutant strains do not colonize mice as well as VacA-producing strains, and the mutant strains exhibit a competitive disadvantage in mixed infections with VacA-producing strains [107,142,144].

*H. pylori* strain SS1, a strain commonly used for experiments in mouse models, contains a non-toxigenic *vacA* allele (s2/i2/m2). SS1 *vacA* null mutant strains exhibit a colonization defect when compared to the wild-type strain [107,142,144]. In one study, SS1 variants producing s1-i2 or s1-i1 forms of VacA exhibited reduced colonization rates compared to strains producing an s2-i2 form of VacA [107]. Thus, despite the lack of detectable activity in vitro, type s2 VacA proteins appear to have an important activity in vivo that contributes to colonization or persistence.

The mechanisms by which VacA contributes to *H. pylori* colonization are not yet well understood, but several hypotheses are plausible. VacA proteins tethered to the surface of *H. pylori* might act as adhesins to promote bacterial adherence to gastric cells, and thereby enhance colonization [148]. VacA-induced alterations of gastric epithelial cells could potentially modify the gastric environment to promote colonization and bacterial replication [65]. VacA-induced inhibition of parietal cell function might facilitate *H. pylori* colonization of the stomach [149,150]. Finally, VacA can attenuate the functions of many types of immune cells [3,4,5,10,11,151,152,153,154], so immunomodulatory actions of VacA might facilitate colonization.

## 6. Role of VacA in Gastric Cancer and Gastric Pathology in Animal Models

Mouse models, gnotobiotic piglets, and the Mongolian gerbil model of *H. pylori* infection have been used to evaluate a potential role of VacA in gastric pathology and carcinogenesis. Mice, piglets, and gerbils each develop a gastric mucosal inflammatory response in response to *H. pylori*. *H. pylori*-induced gastric inflammation is relatively mild in wild-type mice, and *H. pylori*-infected wild-type mice do not develop gastric cancer. *H. pylori*-infected gerbils develop more extensive gastric pathology than mice, including severe gastric inflammation, parietal cell loss and hypochlorhydria, dysplasia, and gastric adenocarcinoma [147,155,156]. The carcinomas in gerbils exhibit some characteristics similar to gastric adenocarcinoma in humans, such as penetration through the muscularis mucosa into the submucosa, but in contrast to gastric cancer in humans, the lesions in gerbils remain relatively small in size and are not known to metastasize. *H. pylori*-infected gerbils do not develop intestinal metaplasia or gastric atrophy (two common precursors of gastric cancer in humans). Thus, the gerbil model of *H. pylori* infection recapitulates several features of gastric carcinogenesis in humans, but some features of the gerbil model differ from features of gastric adenocarcinoma in humans.

One approach for studying the effects of VacA in vivo has been to administer the purified VacA protein or VacA-containing *H. pylori* extracts directly into the stomach of animal models. These studies concluded that VacA can damage the gastric mucosa of mice and stimulate the recruitment of inflammatory cells [18,157,158,159].

A more physiologic approach has entailed the infection of animals with viable *H. pylori* and a comparison of wild-type and *vacA* mutant strains. In experiments with gnotobiotic piglets, no differences in the severity of gastric inflammation were detected when comparing animals colonized with a wild-type strain or a *vacA* null mutant [140]. Similar results were reported in experiments with mice [142], but a subsequent study detected stronger Th1 and Th17 responses and more severe pathology in mice colonized with a *vacA* null mutant strain, compared to the wild-type strain [144]. To compare the activities of different forms of VacA, one study infected mice with strain SS1 variants encoding different forms of VacA [107]. At three weeks post-infection, mice infected with a strain encoding the s1/i1 form of VacA exhibited a significantly greater degree of spasmolytic polypeptide expressing metaplasia (SPEM) than mice infected with a strain encoding the s2/i2 form of VacA [107]. There was also a trend toward higher levels of gastric inflammation in mice infected with strains producing s1/i1 forms of VacA compared to s1/i2 or s2/i2 forms of VacA [107].

No differences in the severity of gastric inflammation have been detected when comparing gerbils colonized with a wild-type strain or a *vacA* mutant strain for time periods of three months to 62 weeks [141,143]. However, at 62 weeks post-infection, animals infected with the wild-type strain had a higher incidence of gastric ulceration compared to animals infected with the *vacA* mutant strain [141]. One *H. pylori* strain commonly used for studies of gastric cancer in the gerbil model (strain 7.13) does not produce a detectable VacA protein [145,146,147]. Therefore, VacA is not required for gastric carcinogenesis in the gerbil model.

## 7. Integrating Results of Human Epidemiologic Studies with Results of Experiments in Animal Models

Many human epidemiologic studies have detected an association between *H. pylori* strains containing certain types of *vacA* alleles (encoding forms of VacA that are active in cell culture models) and an increased risk of gastric cancer or premalignant gastric lesions. In contrast, VacA is not required for the development of gastric cancer in the gerbil model. There are multiple possible explanations for this apparent discrepancy.

One interpretation is that the human epidemiologic results simply reflect the association between certain *vacA* allelic variants and other strain-specific genetic elements that contribute to gastric cancer pathogenesis (e.g., the *cag* PAI or strain-specific genes encoding certain OMPs), and VacA has no direct role in the pathogenesis of gastric cancer. An alternate interpretation is that the rodent models used thus far do not accurately reproduce pathologic events leading to the development of gastric cancer in humans. In support of this latter interpretation, there are known differences in the susceptibility of human CD4+ T-cells and mouse CD4+ T-cells to VacA [38,160]. VacA binds to human CD4+ T-cells and inhibits the activation-induced proliferation of these cells; in contrast, VacA binds at significantly lower levels to murine CD4+ T-cells than human CD4+ T-cells, and does not inhibit the activation-induced proliferation of murine T-cells [38,160]. This difference in susceptibility has been attributed to differences in the β2 integrin receptors present on human and mouse T cells [38]. Limitations of rodent models have also been encountered when studying interactions of *H. pylori* outer membrane adhesins with host cell receptors. For example, the outer membrane protein HopQ binds to CEACAM1 on the surface of human cells, but not to a mouse CEACAM1 orthologue or to any CEACAM receptors produced in gastric tissue from Mongolian gerbils [161,162].

## 8. Mechanisms by which VacA may Influence Gastric Cancer Risk

**Figure 2:**
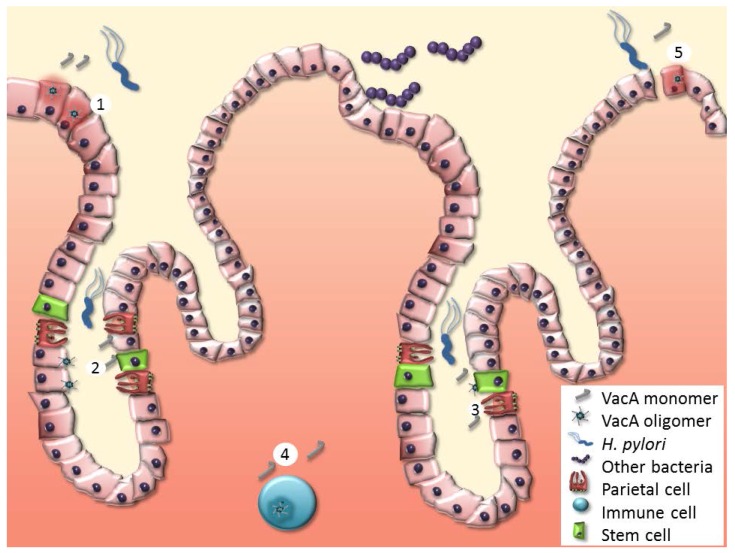
Sites of VacA action relevant to gastric cancer pathogenesis. *H. pylori* colonizes the mucus layer overlying foveolar/surface mucous epithelium and also enters gastric glands. (1) VacA causes multiple alterations in foveolar gastric epithelial cells. (2) Targeting of gastric stem cells by VacA may be a critical step in the pathogenesis of gastric cancer. (3) VacA inhibits acid secretion by parietal cells. Increased gastric pH allows other bacterial species to colonize the stomach. (4) VacA interferes with the function of multiple types of immune cells, potentially compromising their ability to function effectively in surveillance for malignant cells. (5) As a consequence of VacA targeting epithelial cells, tight junctions between gastric epithelial cells are disrupted. This potentially allows carcinogenic molecules to enter the gastric mucosa.

There are multiple biologically plausible mechanisms by which specific forms of VacA could enhance gastric cancer risk (Figure 2). Since *H. pylori* binds to gastric epithelial cells in vivo, these cells probably encounter relatively high concentrations of VacA in vivo. Type s1-m1 forms of VacA promote the death of gastric epithelial cells in vitro [48,69,70,71,72], and the toxin might have similar effects in vivo. VacA-induced death of gastric epithelial cells would be expected to result in increased cellular proliferation, which could be associated with increased cancer risk. VacA has been reported to disrupt the integrity of epithelial monolayers, either by causing cell death or by the loosening of cell-cell junctions [163,164]. Consequently, VacA might also enhance the entry of carcinogens into the gastric mucosa, or may enhance the invasiveness and spread of malignant cells.

Connexin 43 (Cx43) is required for VacA-induced necrosis of the AZ-521 cell line (recently reported to be a misidentified cell line of HuTu-80, human duodenum carcinoma) [165,166]. Cx43 is a tumor suppressor in multiple cell types, and gastric cancers frequently exhibit a loss of Cx43 expression [167]. Therefore, in individuals infected with *H. pylori* strains producing high levels of s1-i1-m1 VacA, there may be a selective pressure for the emergence of Cx43-deficient cells (resistant to VacA-induced cell death), which could contribute to gastric cancer pathogenesis.

Most *H. pylori* localize within the mucus layer overlying foveolar surface mucous epithelial cells, but *H. pylori* can also enter the gastric glands [168,169]. Within gastric glands, *H. pylori* localizes in close proximity to gastric stem cells, and within the oxyntic glands of the gastric corpus, *H. pylori* localizes in close proximity to parietal cells. VacA intoxication of gastric stem cells and parietal cells could potentially have deleterious effects relevant to gastric cancer. In vitro experiments indicate that VacA inhibits the acid-producing capacity of parietal cells [149,150]. The inhibition of parietal cell function by VacA would be expected to result in hypochlorhydria, which could increase gastric cancer risk by allowing the proliferation of nitrate-producing bacterial populations that do not normally grow in the acidic gastric environment.

VacA inhibits the activities of multiple types of immune cells in vitro, including T cells, B cells, dendritic cells, eosinophils, mast cells, macrophages, and neutrophils [3,4,5,10,11,151,152,153], and VacA immunomodulatory activity has been detected in vivo [144,170,171]. VacA-induced alterations in immune function could potentially result in impaired tumor surveillance. VacA is also reported to have pro-inflammatory activity [18,153,158,159,172]. Inflammation is a well-known promoter of carcinogenesis [173], so VacA pro-inflammatory activity could contribute to gastric cancer pathogenesis.

## 9. Summary

In summary, numerous epidemiologic studies have shown that *H. pylori* strains containing specific *vacA* allelic types (encoding forms of VacA that are active in cell culture models) are associated with increased gastric cancer risk, and there are multiple biologically plausible mechanisms by which VacA may contribute to gastric carcinogenesis. Conversely, there is relatively little direct evidence in animal models demonstrating a role of VacA in the pathogenesis of gastric cancer. In future studies, it will be important to investigate the actions of VacA in vivo using animal models that are optimized to express cell types susceptible to VacA and that closely replicate the cascade of events leading to gastric adenocarcinoma in humans.

## Figures and Tables

**Table 1:** Association of specific *vacA* allelic types with gastric cancer risk.

*vacA* Allele	Odds Ratio for Developing GC ^a^	Location	Reference
s region			
s1	17 (7.8–38)	Portugal	Figueiredo, 2002 [98]
s1	8.3 (2.8–25)	Italy	Basso, 2008 [99]
s1	5.6	Iran	Rhead, 2007 [81]
i region			
i1	5.0 (2.1–12)	Italy	Basso, 2008 [99]
i1	8.7	Iran	Rhead, 2007 [81]
m region			
m1	6.7 (3.6–12)	Portugal	Figueiredo, 2002 [98]
m1	5.3 (1.0–27)	Italy	Basso, 2008 [99]
m1	3	Iran	Rhead, 2007 [81]

**^a^** The Odds Ratio for developing gastric cancer compares the likelihood of gastric cancer occurrence among individuals infected with *H. pylori* strains harboring s1, i1, or m1 alleles vs strains harboring s2, i2, or m2 alleles. The 95% confidence interval is shown in parentheses where available.
